# Systemic Manifestations and Complications in Patients with Rheumatoid Arthritis

**DOI:** 10.3390/jcm9062008

**Published:** 2020-06-26

**Authors:** Ji-Won Kim, Chang-Hee Suh

**Affiliations:** Department of Rheumatology, Ajou University School of Medicine, Suwon 16499, Korea; jwk722@naver.com

Rheumatoid arthritis (RA) is a systemic autoimmune disease with symmetrical peripheral polyarthritis, predominantly involving the small joints. The typical manifestations of RA include pain in one or more joints over several weeks to months and morning stiffness lasting for more than 1 h, which usually improves with exercise. With the increasing recognition of RA and development of novel treatment strategies, early diagnosis and prompt treatment may result in some patients achieving remission status or low disease activity; however, many patients do not receive adequate treatment or respond to treatment. When not adequately treated at an early stage, the chronic inflammation caused by RA results in joint destruction, which can lead to disability, lower functional status, early unemployment, and a reduced life expectancy. The expected survival of RA patients is likely to be decreased by 3–10 years compared with that of the general population [1]. The decreased life expectancy is associated with various systemic manifestations and complications related to the treatment. Systemic manifestations and complications of RA—including pulmonary, cardiovascular, neurological, and musculoskeletal involvements; glucocorticoid (GC)-induced osteoporosis (GIOP); and infection—which have significant impacts on the disease outcomes, occur in approximately 40% of patients [2]. The risk factors for both are not only severe inflammation and a long-standing disease duration but also seropositivity (rheumatoid factor and anti-cyclic citrullinated antibodies), smoking, and male sex [3]. RA patients with systemic manifestations or complications should be treated and monitored more aggressively to reduce their risk of mortality. This paper will briefly review the systemic manifestations and complications of RA.

## 1. Pulmonary Manifestations

In 1948, lung disease in association with RA was first described, and since then, various lung involvements such as pleural disease, parenchymal disease, pulmonary nodules, airway disease, and vasculitis have been reported [4,5]. Pulmonary involvement accounts for a high proportion of systemic manifestations, ranging from 60% to 80%, which is increased by either opportunistic infections associated with immunosuppressant use or direct pulmonary toxicity from methotrexate and anti-tumor necrosis factor agents [5]. Among the various pulmonary manifestations, interstitial lung disease (ILD) is known to be associated with substantial morbidity and mortality rates in RA patients. As RA-ILD is often asymptomatic, its prevalence is variable and ranges from 19% to 67%. Among patients, approximately 10% develop a clinically significant disease, defined as a contributing factor for death, and the median survival is 5–8 years after symptomatic ILD diagnosis [6]. Unfortunately, the treatment options for RA-ILD are still complicated and unclear because of the possible pulmonary toxicity of disease-modifying anti-rheumatic drugs (DMARDs) and their unclear effectiveness for lung diseases. Cyclophosphamide and mycophenolate mofetil showed little efficacy in the treatment of RA-ILD, and several clinical trials are ongoing to demonstrate the efficacy and safety of antifibrotic agents [7]. Pleural diseases are also common intrathoracic manifestations of RA, although most patients are asymptomatic over a lifetime. Nevertheless, in some pleural diseases, complications requiring repeated intervention occur—such as secondary pneumothorax, empyema, abscesses, and bronchopleural fistula formation—and may be resistant to standard therapies. The well-known airway diseases in RA patients are bronchiolitis and bronchiectasis, occurring in 10–30% of patients. Persistent inflammation caused by a dysregulated immune response in RA occurs in the airway, occasionally destroying the peripheral airway or forming honeycomb-like structures in the lung parenchyma [8]. Rheumatoid pulmonary nodules are generally asymptomatic, often multiple, and varying in size from a few millimeters to several centimeters, and the prognosis is mostly good, with spontaneous resolution and few complications. 

## 2. Cardiovascular Manifestations

Cardiac and vascular manifestations of RA vary widely, including cardiovascular disease (CVD), heart failure, arrhythmia, valve disease, pericarditis, and myocarditis. Among the various diseases, CVD is the leading cause of mortality in RA, with a mortality rate 1.5- to 3.0-fold higher than that in the general population [9]. Traditional risk factors that accelerate atherosclerosis—such as smoking, high blood pressure, and hyperlipidemia—are important but insufficient for explaining the full extent of CVD risk. Genetic factors, oxidative stress, and therapeutic agents including non-steroidal anti-inflammatory drugs or GCs have also been found to be independent risk factors contributing to endothelial dysfunction and vascular damage. On the other hand, conventional DMARDs such as methotrexate, hydroxychloroquine, and sulfasalazine and most biologic DMARDs (bDMARDs) have been shown to improve CV risk by regulating chronic inflammation. The routine assessment of CVD risk and selection of anti-rheumatic drugs to lower disease activity are essential steps for minimizing CVD risk. In addition, increasing evidence indicates that occurrences of congestive heart failure, atrial fibrillation, and valvular thickening have become more frequent in RA patients [10]. Among the non-atherosclerotic cardiac manifestations of RA, pericarditis is one of the most common. According to the results of the echocardiography or post-mortem examinations of RA patients, pericardial inflammation was found in 30–50% of patients, although only 1–4% of them showed symptoms [11]. Endocarditis, myocarditis, and amyloidosis are relatively rare complications of RA. 

## 3. Neurological Manifestations

Neurological abnormalities in RA encompass the peripheral (PNS) or central (CNS) nervous systems, with symptoms ranging from sudden death to mild paresthesia. Most complications are a consequence of articular inflammation that compresses or invades the adjacent spinal cord, peripheral nerve, or neural tissues. Disorders of the CNS include cervical myelopathy, vasculitis, rheumatoid meningitis, rheumatoid nodules located within the CNS, and progressive multifocal leukoencephalopathy. Cervical myelopathy is the most common manifestation of CNS, reported in up to 50% of RA patients [12]. It results from arthritis of the atlantoaxial joint and the subsequent erosion of the odontoid process. Occasionally, impingement of the medulla can lead to sudden death; thus, surgical intervention is indicated in suspected cases. Though uncommon, other CNS involvements may result in massive bleeding, infarction, or quadriplegia. Stroke and autonomic dysfunction tend to increase in frequency in RA and are related to higher disease activity. PNS involvement is present in approximately 30% of RA patients and includes both compressive (entrapment) and non-compressive neuropathies [13]. Carpal tunnel syndrome, which compresses the median nerve, is the most common entrapment neuropathy and is correlated with the severity and degree of local synovitis. Non-compressive neuropathies are caused by small vessel vasculitis and manifest as mononeuritis multiplex, distal sensory neuropathy, and sensorimotor neuropathy. Furthermore, the prevalence of severe demyelinating diseases such as Guillain–Barré syndrome induced by biologic DMARDs has increased in recent years owing to the widespread use of these agents. 

## 4. Musculoskeletal Involvement

RA-associated systemic and local inflammation induces many changes in skeletal health. In early disease, periarticular osteopenia and juxta-articular bone erosions occur adjacent to inflamed and swollen joints. High disease activity and a long disease duration can lead to joint ankylosis. It is widely agreed that reducing disease activity using conventional or biologic DMARDs during the early phase may retard or prevent the progression of bone erosions. Chronic inflammation caused by RA that is not adequately treated at an early stage or does not respond to treatment can induce joint fusion, generalized bone loss, osteoporosis, and fractures. The prevalence of osteoporosis was 1.5- to 2-fold higher in RA patients than in age- and sex-matched subjects from the general population [14]. The development of osteoporosis increases the incidence of femoral neck and vertebral compression fractures, leading to a further decrease in quality of life and increased mortality. Within 6 months of the onset of fractures, approximately 20–30% of RA patients with osteoporosis die of complications caused by prolonged immobilization and postoperative complications [15]. In addition to disease duration and activity, GC therapy, immobility, a low body mass index, sarcopenia, and opioid use are strongly associated with fracture risk. This means that clinicians can prevent osteoporosis and fractures by reducing the use of drugs that promote bone loss, regularly assessing fracture risk using bone mineral density or a fracture risk assessment tool, evaluating falls, and recommending vitamin D and calcium supplements. Unfortunately, although GCs are recognized to play a crucial role in the development of osteoporosis, the use of GCs in RA treatments is more common than in any other inflammatory disease. GCs have the advantage of rapid action, are often necessary to control acute inflammation, and are safe for pregnancy and breastfeeding. In this regard, achieving a remission status by administering low-dose GCs over a short period has been suggested to ultimately prevent bone erosion; however, the safety of GCs with minimal toxicity remains controversial because even low doses of GCs can cause GIOP with prolonged use.

Muscle disorders in RA patients include myopathy and myositis and have been mostly attributed to active diseases, increased body fat mass, reduced lean body mass (sarcopenia), and immobilization. Although they may be caused by the loss of muscle mass linked to chronic inflammation, muscle weakness commonly occurs due to pharmacotherapies such as GCs, hydroxychloroquine, and lipid-lowering agents. Thus, drugs should be examined and considered as causes of RA in patients who complain of muscle weakness or myalgia.

## 5. Infection

RA patients are widely known to have a higher risk of infection than the general population, and serious infection is one of the main causes of death in RA. The lower respiratory system is the most commonly involved site, and the other frequently involved sites are the skin, soft tissues, bloodstream, bones, joint, and urinary tract. The risk of tuberculosis or opportunistic infections also appears to be higher in RA patients. The increased risk of infections may be explained by the intrinsic immunological disturbances of the disease itself, comorbidities, old age, and the iatrogenic effects of therapeutic agents. The use of GCs, especially in high doses, and immunosuppressive drugs inhibits the immune response, delays the clearance of pathogens, and suppresses the release of host inflammatory cytokines, making infections a central concern. In the early development of bDMARDs, a high risk of infection was consistently reported; however, data on the risk of infection associated with bDMARDs have been discrepant in the recent literature. An analysis of recent data from a Danish biologics registry of patients hospitalized with and treated for pneumonia revealed that the mortality rate in RA patients was not higher than that in patients without RA, and only high disease activity, regardless of therapeutic agents, increased the mortality rate [16]. Despite the release of new studies contradicting the existing results, the prevailing view is that the use of immunosuppressive drugs increases the risk of infection, and in severe infections, these agents should be temporarily suspended until the resolution of the infection. Therefore, clinicians should measure the associated potential risk of infection while targeting and maintaining remission or low disease activity with aggressive treatment strategies in RA. The relationship between RA and infection is a challenge that rheumatologists must permanently solve with the development of immunosuppressants.

## 6. Conclusions

The development of the pharmaceutical industry in relation to RA has greatly improved disease outcomes, and the incidence of symptoms associated with chronic inflammatory reactions and established comorbidities has decreased correspondingly. However, the management of systemic manifestations and complications remains a challenge, as it has not equally improved in all areas, and new complications have emerged owing to the many targeted therapies. Therefore, rheumatologists play a pivotal role in the prevention of, approaches to, and management of these complications and in coordinating care among other healthcare providers. We look forward to future studies on the diagnostic, preventive, and treatment aspects of various complications in RA patients.
